# Unraveling *in vitro* phase separation and aggregation properties of the structured region of FMRP and the impact of Fragile X syndrome‐linked mutations

**DOI:** 10.1111/febs.70564

**Published:** 2026-05-02

**Authors:** Flavia Catalano, Alessandra Bigi, Francesca Troilo, Federica Gabriele, Gianluca Pistoia, Italia A. Asteriti, Francesco Angelucci, Giorgio Giardina, Fabrizio Chiti, Carlo Travaglini‐Allocatelli, Adele Di Matteo

**Affiliations:** ^1^ Institute of Molecular Biology and Pathology National Research Council of Italy Rome Italy; ^2^ Department of Experimental and Clinical Biomedical Sciences, Section of Biochemistry University of Florence Florence Italy; ^3^ Department of Health, Life and Environmental Sciences University of L'Aquila Italy; ^4^ Department of Biochemical Sciences “A Rossi Fanelli”, Sapienza University of Rome Italy

**Keywords:** amyloid fibrils, Fragile X syndrome, KH domains, liquid–liquid phase separation

## Abstract

Fragile X messenger ribonucleoprotein 1 (FMRP) is a multidomain RNA‐binding protein highly expressed in neurons. It comprises a structured N‐terminal region (NTR) containing five RNA/protein interaction domains and a large intrinsically disordered C‐terminal region. A limited number of fragile X syndrome (FXS)‐associated point mutations have been identified in the NTR, leading to the expression of mutated protein variants. Here, we show that the destabilizing effects of these mutations on the NTR region are less severe than those previously observed in the isolated domains, suggesting the presence of inter‐domain interactions that stabilize the overall NTR architecture. Moreover, we demonstrate that the NTR structured region has an intrinsic propensity to undergo liquid–liquid phase separation (LLPS) and amyloid fibril formation *in vitro*, depending on protein concentration, and we characterize how three FXS‐associated mutations (R138Q, G266E, and I304N) affect these processes. We show that the mutations either destabilize the entire NTR (G266E/I304N), markedly affect the kinetics of LLPS and suppress the droplet liquid nature (R138Q/G266E), or promote solid or gel‐like non‐amyloid aggregation (G266E), thus providing mechanistic insights into how they may alter protein function, contributing to the pathogenic mechanism. These findings suggest that the interplay between protein stability, LLPS, and fibrillization is finely regulated and may be critical for understanding FMRP function and its dysfunction in disease.

## Graphical abstract

Fragile X messenger ribonucleoprotein 1 (FMRP) is a multidomain RNA‐binding protein associated with Fragile X Syndrome (FXS). We found that its N‐terminal structured region has an intrinsic propensity to undergo liquid–liquid phase separation and fibril formation. FXS‐associated mutations perturb protein stability and aggregation propensity, suggesting that altered condensation behavior may be critical for FMRP dysfunction in disease.

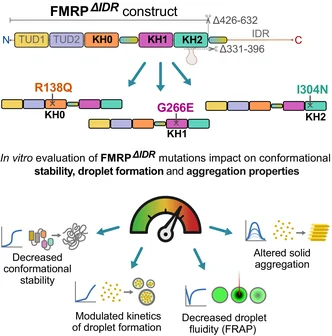

**Abbreviations**
CD: circular dichroism
CTR: C‐terminal region
DIC: differential interference contrast
DTT: dithiothreitol
FMRP: Fragile X messenger ribonucleoprotein 1
FRAP: fluorescence recovery after photobleaching
FXS: Fragile X syndrome
IDR: intrinsically disordered region
KH: K‐homology domain
LLPS: liquid–liquid phase separation
NTR: N‐terminal region
PEG: polyethylene glycol
RGG: arginine‐glycine–glycine motif
TEM: transmission electron microscopy
ThT: thioflavin T

## Introduction

Fragile X messenger ribonucleoprotein 1 (FMRP) is a nuclear‐cytoplasmic RNA‐binding protein (RBP), highly expressed in the brain and in the reproductive system [1] and is identified as a key regulator of synaptic plasticity and architecture [2]. It is encoded by the *fmr1* gene located on the chromosome locus Xq27.3. The gene consists of 17 exons and 16 introns and its transcript undergoes alternative splicing, thereby generating multiple mature isoforms [3], with the longest one, considered the canonical isoform, composed of 632 residues. All isoforms share the same N‐terminal region, except for the variable loop of one of its domains, while the main differences are located in the intrinsically disordered C‐terminal region [3, 4].

From a structural perspective, FMRP consists of a structured N‐terminal region (NTR) and an intrinsically disordered C‐terminal region (CTR or IDR) which contains a characteristic repeating Arg‐Gly‐Gly (RGG) motif [5]. The NTR includes two Tudor Domains (TD1 and TD2, also known as Agenet domains) paired with an hnRNP K‐homology domain (KH), termed KH0, which is connected via a linker region to two additional KH domains arranged in tandem (KH1 and KH2) (Fig. 1). KH1 and KH2 adopt the canonical KH fold, consisting of a β‐sheet flanked by three α‐helices and featuring a GXXG motif in the loop connecting α1 and α2 [6], which is critical for nucleic acid binding [7], while KH0 lacks the GXXG motif, suggesting an alternative mechanism of RNA recognition. Although the three‐dimensional structures of the TD1–TD2, TD1–TD2–KH0, KH1–KH2, and KH1 constructs, as well as the RGG region in complex with nucleic acids, have been resolved using X‐ray crystallography and NMR, as reviewed in [5], the complete structure of the NTR containing all five structured domains is still lacking and can currently only be predicted by AlphaFold [8] (Fig. 1).

**Fig. 1:**
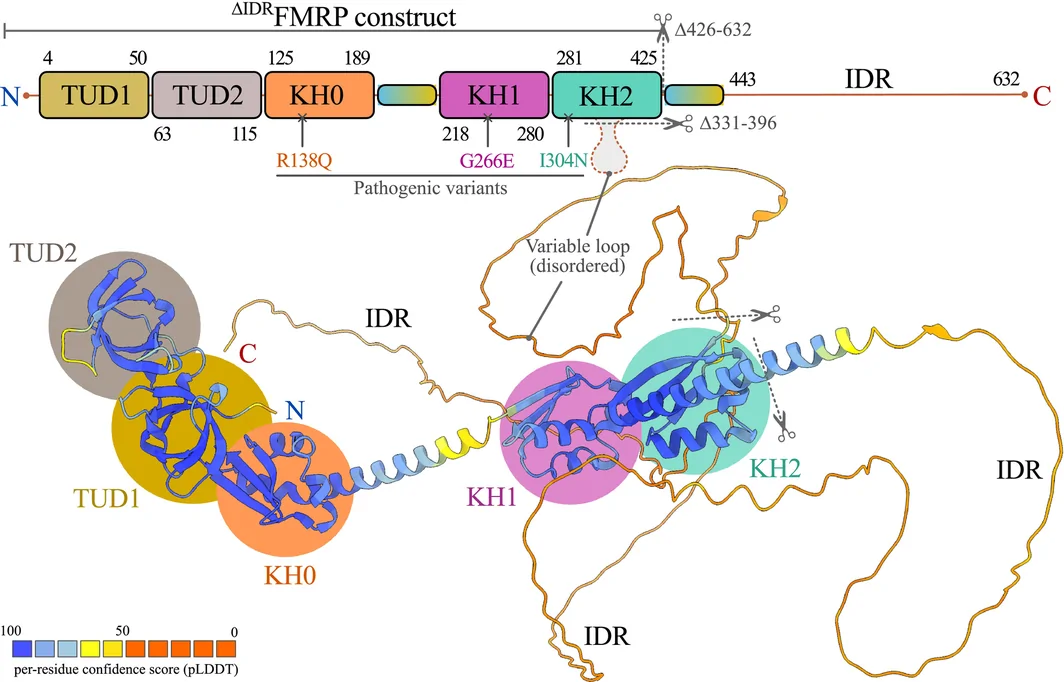
Domain organization and structure prediction of FMRP. Schematic representation of the domain architecture of the canonical human FMRP isoform (UniProt: Q06787‐1) and its AlphaFold model (AF‐Q06787‐F1) colored according to the predicted local distance difference test (pLDDT) score. The recombinant protein construct used in this study (FMRP^ΔIDR^) is highlighted.

The multidomain organization of FMRP enables it to be a pleiotropic protein able to interact with both other proteins [9, 10, 11] and various forms of RNA [12, 13, 14, 15], thereby fulfilling a multiplicity of functions. It is well known as a translational repressor [13, 16, 17, 18, 19] and as a regulator of gene expression through the modulation of transcription, stability, transport, modification, and translation of hundreds of mRNAs involved in neuronal functions [15, 20, 21]. In addition, FMRP is involved in many other cellular functions [10], including control of ion channel expression and activity [22], chromatin dynamics [23, 24], DNA damage response (DDR), and stress response [11].

Moreover, FMRP associates with various ribonucleoprotein (RNP) assemblies, including transport mRNP granules, Fragile X granules (FXGs) together with its two autosomal homologs FXR1 and FXR2, P‐bodies, and stress granules [25]. In neurons, RNA transport granules containing FMRP carry translationally repressed mRNAs to synapses, where they are derepressed in response to synaptic activity [25]. Such granules are part of a class of membraneless organelles (MLOs), formed through a process of liquid–liquid phase separation (LLPS), driven by weak and transient multivalent macromolecular interactions, including both protein–protein and protein–RNA interactions [25]. Several *in vitro* studies have demonstrated the ability of FMRP to undergo LLPS, highlighting the importance of its intrinsically disordered C‐terminal region in this process [26, 27, 28, 29, 30]. Tsang and colleagues initially reported that the low‐complexity region of FMRP alone is necessary and sufficient to drive phase separation *in vitro* in the presence of sc1 RNA, and that post‐translational modifications such as phosphorylation and methylation exert opposite effects on phase separation propensity, which correlates with translational repression [28]. However, more recent findings have highlighted the importance of the N‐terminal region of FMRP and its homolog FXR1 in the formation and dynamics of granules [31, 32, 33, 34].

Given its pleiotropic functions, it is not surprising that FMRP participates in multiple aspects of cellular processes. Alterations in its activity, due to dysfunction or dysregulation, are associated with pathological conditions. Historically, FMRP has been linked to Fragile X syndrome (FXS), the most common inherited form of intellectual disability [35], as well as related disorders such as Fragile X‐associated primary ovarian insufficiency (FXPOI) [36] and Fragile X‐associated tremor/ataxia syndrome (FXTAS) [37]. More recently, FMRP has also been associated with amyotrophic lateral sclerosis (ALS) [38], viral infections [39, 40], and cancer [41, 42, 43]. In 99% of cases, FXS is caused by an abnormal expansion of CGG repeats in the 5′UTR region of the *fmr1* gene, leading to hypermethylation of the *fmr1* locus, gene silencing, and complete absence of the protein [15, 44].

However, a limited number of missense mutations in the *fmr1* gene associated with FXS have also been identified, resulting in the expression of a mutated protein. Among these, three well‐characterized are located within the KH domains of FMRP (R138Q in KH0, G266E in KH1, and I304N in KH2) and alter the protein functions, although their precise contribution to the pathological phenotypes remains largely unclear [45, 46]. These mutations can be leveraged to gain deeper insight into the contribution of individual KH domains to FMRP function and pathogenicity.

In this study, we investigated the role of the NTR of FMRP in *in vitro* condensation propensity and compared the effects of the three FXS‐associated mutations located in the KH domains (R138Q, G266E, and I304N) on this process. Moreover, given the growing body of evidence suggesting that liquid condensate formation contributes to the development of stable and organized aggregates, such as amyloid fibrils [47, 48], as observed for α‐synuclein [49], Tau [50], and the TDP‐43 C‐terminal region [51] and considering that FXR proteins tend to oligomerize and aggregate [10, 52, 53, 54], we explored the propensity of the FMRP NTR and its variants to form solid aggregates. Our results show a concentration‐dependent ability of the NTR of FMRP to form either liquid droplets through LLPS or fibrillar aggregates, behaviors that are differentially modulated by the FXS‐associated mutations. These findings indicate that a delicate balance exists between LLPS and fibrillar aggregation for FMRP, and that the structured portion of the protein, in addition to its disordered region, plays a key role in these processes.

## Results

### *In vitro* phase separation propensity of the FMRP structured region

To investigate the ability of the structured NTR of FMRP (residues 1–425, hereafter FMRP^ΔIDR^) to undergo phase separation, we expressed in *E. coli* a construct comprising all the structured Tudor and KH domains, excluding the intrinsically disordered region (IDR; residues 426–632) and the variable loop within KH2 (residues 331–396, based on the three‐dimensional structure of the KH1KH2 tandem [55]) (Fig. 1).

To evaluate the propensity of FMRP^ΔIDR^ to phase separate *in vitro* and to define the minimal protein concentration required for this process to occur, we firstly performed a turbidimetry assay [56] as a function of protein concentration in the presence of 10% polyethylene glycol 8 K (PEG 8 K), a common additive used to mimic the intracellular crowding conditions [57], in 20 mm Tris/HCl (pH 7.4), 100 mm NaCl, 2 mm DTT (hereafter referred to as self‐assembly buffer) at room temperature (RT). As shown in Fig. 2A, an increase in turbidity is evident starting from a critical threshold FMRP^ΔIDR^ concentration of ~2.5 μm (apparent C*sat*). Since both aggregation and LLPS can contribute to turbidity [58, 59], we used additional techniques to characterize the nature of the assemblies formed under these conditions. Differential interference contrast (DIC) microscopy at 2.5 μm protein at RT, revealed the presence of round assemblies (Fig. 2B). These species were further confirmed using FMRP^ΔIDR^ at 3 μm labeled with the AlexaFluor 488 dye (1:10 molar ratio of dye:protein) by confocal fluorescence microscopy. Immediately after inducing phase separation, the images revealed the formation of round‐shaped condensates with an average diameter of 0.3–1.0 μm (Fig. 2C). Since droplets were clearly observed by DIC microscopy using unlabeled FMRP^ΔIDR^ at similar concentration (Fig. 2B), we can conclude that the fluorophore does not influence the phase separation propensity of FMRP^ΔIDR^. Fluorescence recovery after photobleaching (FRAP) experiments were then carried out to investigate the internal molecular dynamics of these species at the same FMRP^ΔIDR^ concentration of 3 μm (Fig. 2F,G). The result revealed a rapid (~20 s) and nearly complete (~95%) recovery of the bleached fluorescence, confirming the liquid‐like nature of these condensates. A similar behavior was also observed at 5 μm protein (Fig. 2D), as the state of matter of these species was still liquid (Fig. 2F,G). By contrast, at 10 μm FMRP^ΔIDR^, large solid aggregates (Fig. 2E) with no fluorescence recovery were observed (Fig. 2F,G).

**Fig. 2:**
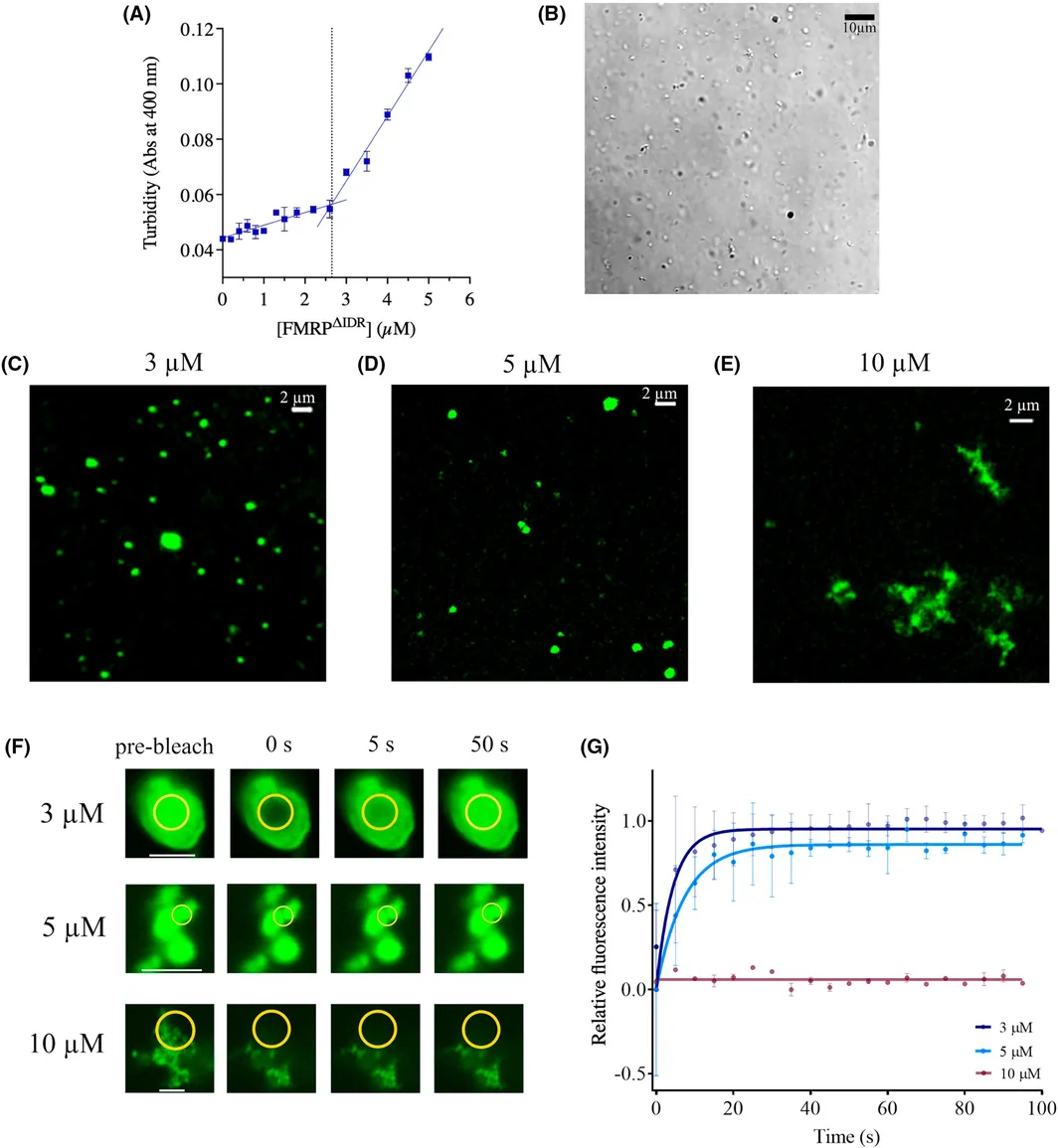
*In vitro* phase separation propensity of FMRP^ΔIDR^. (A) Turbidity (OD at 400 nm) of WT‐FMRP^ΔIDR^ in self‐assembly buffer as a function of protein concentration. The concentrations at which the two straight lines fitted through the experimental data intersect correspond to the apparent Csat, highlighted by the dotted line. Error bars represent SD (*n* = 3). (B) Representative (from *n* = 3 independent experiments) DIC microscopy images obtained using 2.5 μm unlabeled WT‐FMRP^ΔIDR^ in self‐assembly buffer. (C–E) Representative confocal fluorescence microscopy images of Alexa Fluor 488‐labeled WT‐FMRP^ΔIDR^ at 3 μm (*n* = 5) (C), 5 μm (*n* = 5) (D), 10 μm (*n* = 3) (E) in self‐assembly buffer after mixing. (F) Representative confocal microscopy images at different time points from the FRAP experiments of WT‐FMRP^ΔIDR^ at 3 μm, 5 μm and 10 μm protein. Scale bars: 2 μm. (G) FRAP recovery curves of FMRP^ΔIDR^ at 3 μm (dark blue line), 5 μm (light blue line), 10 μm (purple line). Averaged assemblies were analyzed (*n* = 7 for 3 μm; *n* = 2 for 5 μm; *n* = 2 for 10 μm). Relative fluorescence intensity post‐photobleaching was plotted over time; fluorescence values were normalized to pre‐bleach (taken as 1) and post‐bleach (taken as 0). Error bars: SD. Yellow circles indicate the approximate bleached area; bleaching Regions of Interest (ROIs) were defined during acquisition and adapted to sample morphology.

Collectively, these data demonstrate that the NTR of FMRP has an intrinsic propensity to undergo LLPS in a crowded environment at pH 7.4 and RT and that the formation of liquid‐like or solid self‐assemblies is a protein concentration‐dependent process.

### FXS‐linked mutations affect the stability of FMRP^ΔIDR^

The point mutations R138Q, G266E, and I304N, located in the KH0, KH1, and KH2 domains of FMRP, respectively (Fig. 1), are associated with FXS [46]. Previous studies on isolated KH domains carrying these substitutions have reported distinct effects on domain conformational stability and propensity to form fibrillar aggregates [60, 61, 62].

Here, we investigated the effect of these mutations on the entire structured NTR of the protein by introducing the three substitutions individually into FMRP^ΔIDR^ and studying the resulting recombinant constructs (R138Q‐FMRP^ΔIDR^, G266E‐FMRP^ΔIDR^, and I304N‐FMRP^ΔIDR^). As shown in Fig. 3A, the far‐UV CD spectra of WT‐FMRP^ΔIDR^ and its three FXS‐linked variants are all consistent with properly folded α/β proteins. These results indicate that these mutations do not significantly perturb the overall secondary structure of FMRP^ΔIDR^, with only the G266E substitution resulting in a minor increase in disordered content, as evidenced by a slight shift of the 208 nm minimum toward lower wavelengths.

**Fig. 3:**
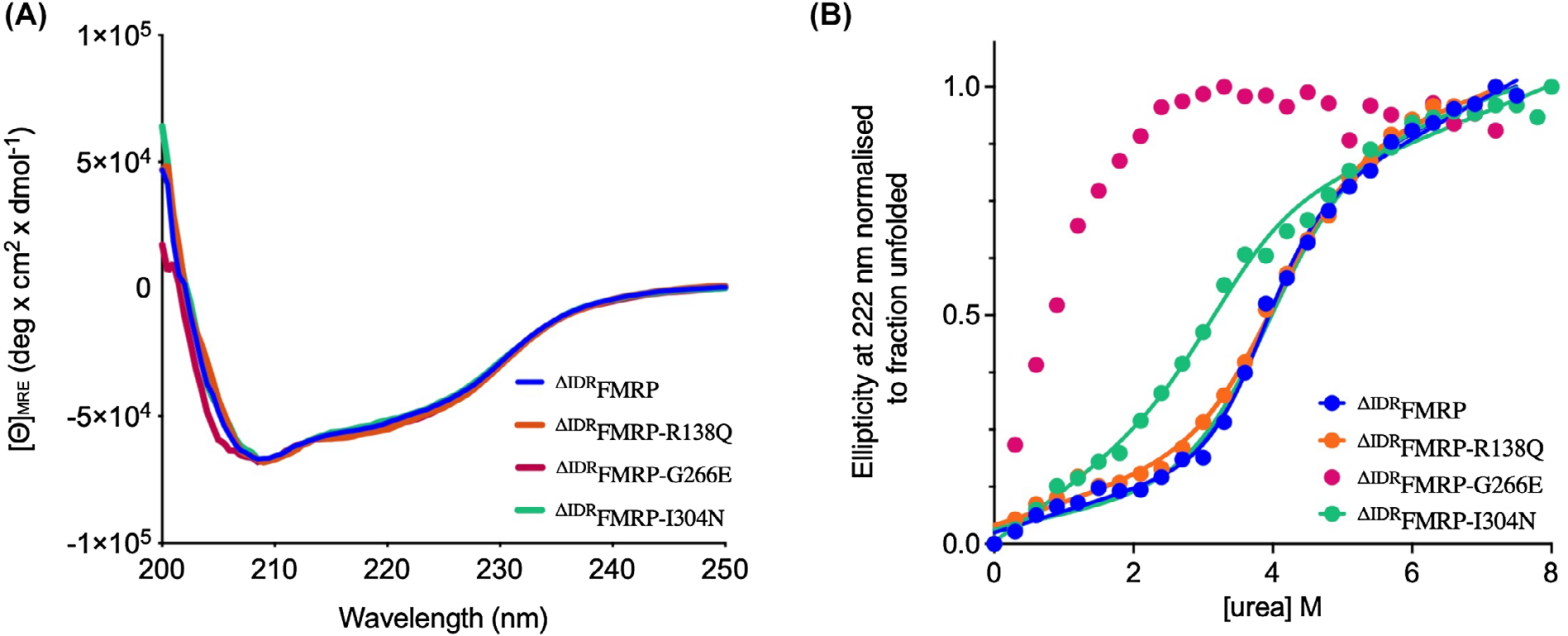
Secondary structure and stability of WT‐ FMRP^ΔIDR^ and FXS‐linked variants. (A) Far‐UV CD spectra recorded at 20 °C in 20 mm Tris/HCl (pH 7.4), 100 mm NaCl. (B) Urea‐induced denaturation monitored by far‐UV CD. Experiments were performed in 20 mm Tris/HCl (pH 7.4), 100 mm NaCl, at 20 °C. Ellipticity at 222 nm was plotted as a function of urea concentration and data were normalized to the fraction of unfolded protein. Where applicable, data were fitted with Eq. 1 (see Methods) and the resulting thermodynamic parameters are reported in Table 1.

Figure 3B shows the urea‐induced denaturation curves, monitored by far‐UV CD, for all multidomain constructs, while the thermodynamic parameters obtained from curve fitting (see Materials and Methods) are reported in Table 1. Our analysis revealed that the conformational stability (Δ*G*_*D‐N*_) and unfolding cooperativity (*m* value) of R138Q‐FMRP^ΔIDR^ are very similar to those of the WT construct, in agreement with previous results on the isolated KH0 domain, where the R138Q mutation was shown to preserve the structural integrity of the domain, causing only a slight destabilization [62]. Interestingly, this R138Q‐induced destabilization appears lower, if any, on the FMRP^ΔIDR^ construct studied here rather than on the isolated KH0 domain. In contrast to the R138Q mutation, a clear destabilization (ΔΔ*G*_*D‐N*_ ~ 3.7 Kcal mole^−1^) along with a less cooperative denaturation process (lower *m* value) was observed for I304N‐FMRP^ΔIDR^ (Fig. 3B, Table 1). An even more marked destabilization was observed for G266E‐FMRP^ΔIDR^, in which the absence of a pre‐transition baseline in its denaturation curve precluded reliable curve fitting (Fig. 3B, Table 1). Notably, although the G266E and I304N substitutions were found to unfold the KH1 and KH2 isolated domains ([60, 61] and our unpublished data), the overall secondary structure of G266E‐FMRP^ΔIDR^ and I304N‐FMRP^ΔIDR^ was largely maintained (Fig. 3A), suggesting that the mutation‐induced destabilization does not lead to the unfolding of the multidomain protein construct.

**Table 1:** Thermodynamic parameters obtained by urea‐induced denaturation (Fig. 3B). The *m* value was shared during the fitting procedure for WT‐FMRP^ΔIDR^ and R138Q‐FMRP^ΔIDR^.

	[urea]_1/2_ (M)	*m* (kcal mol^−1^ m ^−1^)	*∆G*_*D‐N*_ (kcal mol^−1^)
WT‐FMRP^ΔIDR^	3.9 ± 0.1	1.2 ± 0.1	4.9 ± 0.1
R138Q‐FMRP^ΔIDR^	4.0 ± 0.1	1.2 ± 0.1	4.8 ± 0.1
G266E‐FMRP^ΔIDR^	n.d	n.d	n.d
I304N‐FMRP^ΔIDR^	2.9 ± 1.0	0.4 ± 0.2	1.2 ± 0.6

Our results show that the FXS‐linked mutations have distinct impacts on the structure and conformational stability of FMRP^ΔIDR^, with the most critical being the G266E substitution. However, the destabilizing effects of these mutations on the FMRP^ΔIDR^ construct are less severe, that is, have a lower Δ*G*_*D‐N*_, than those previously observed for the individual domains, when purified and studied as separate constructs [60, 61, 62], suggesting the presence of inter‐domain interactions that stabilize the overall FMRP^ΔIDR^ architecture and buffer the effects of the mutations.

### FXS‐linked mutations alter the phase separation propensity of FMRP^ΔIDR^

To investigate whether these FXS‐linked mutations affect the propensity of FMRP^ΔIDR^ to undergo LLPS, we first performed turbidimetry analyses in self‐assembly buffer at RT at different protein concentrations. All three mutant constructs display similar apparent saturation concentrations (C*sat* ~ 1–3 μm); however, while clear critical concentrations for phase transition were observed for R138Q‐FMRP^ΔIDR^ and I304N‐FMRP^ΔIDR^, the transition for G266E‐FMRP^ΔIDR^ appeared smoother (Fig. 4). To examine the self‐assembly properties of FMRP^ΔIDR^ variants, we monitored AlexaFluor 488‐labeled proteins (3 μm) by confocal fluorescence microscopy at multiple time points from 1 to 60 min (±1 min), following induction of phase separation in the self‐assembly buffer at RT (Fig. 5A).

**Fig. 4:**
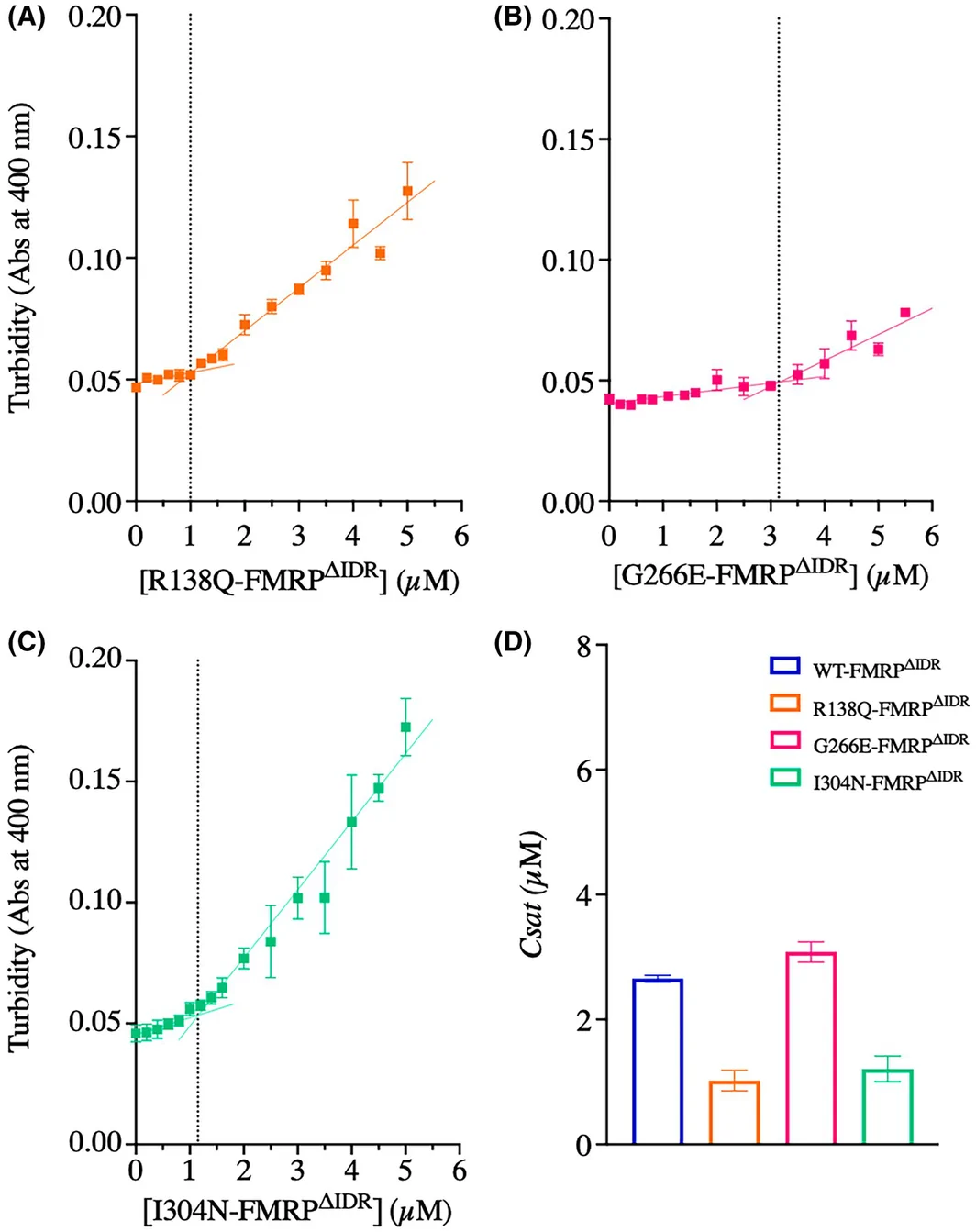
Turbidity‐based analysis of the FMRP^ΔIDR^ FXS‐linked variants. (A–C) Turbidity (OD at 400 nm) measured as a function of protein concentration for R138Q‐FMRP^ΔIDR^ (A), G266E‐FMRP^ΔIDR^ (B), I304N‐FMRP^ΔIDR^ (C) in 20 mm Tris/HCl (pH 7.4), 100 mm NaCl, 2 mm DTT, 10% PEG 8 K at RT. The concentrations at which the two straight lines fitted through the experimental data intersect corresponds to the apparent Csat, highlighted by the dotted line. Error bars represent SD (*n* = 3). (D) Apparent saturation concentrations for FXS‐linked variants. The value of WT‐FMRP^ΔIDR^ is taken from Fig. 2A.

**Fig. 5:**
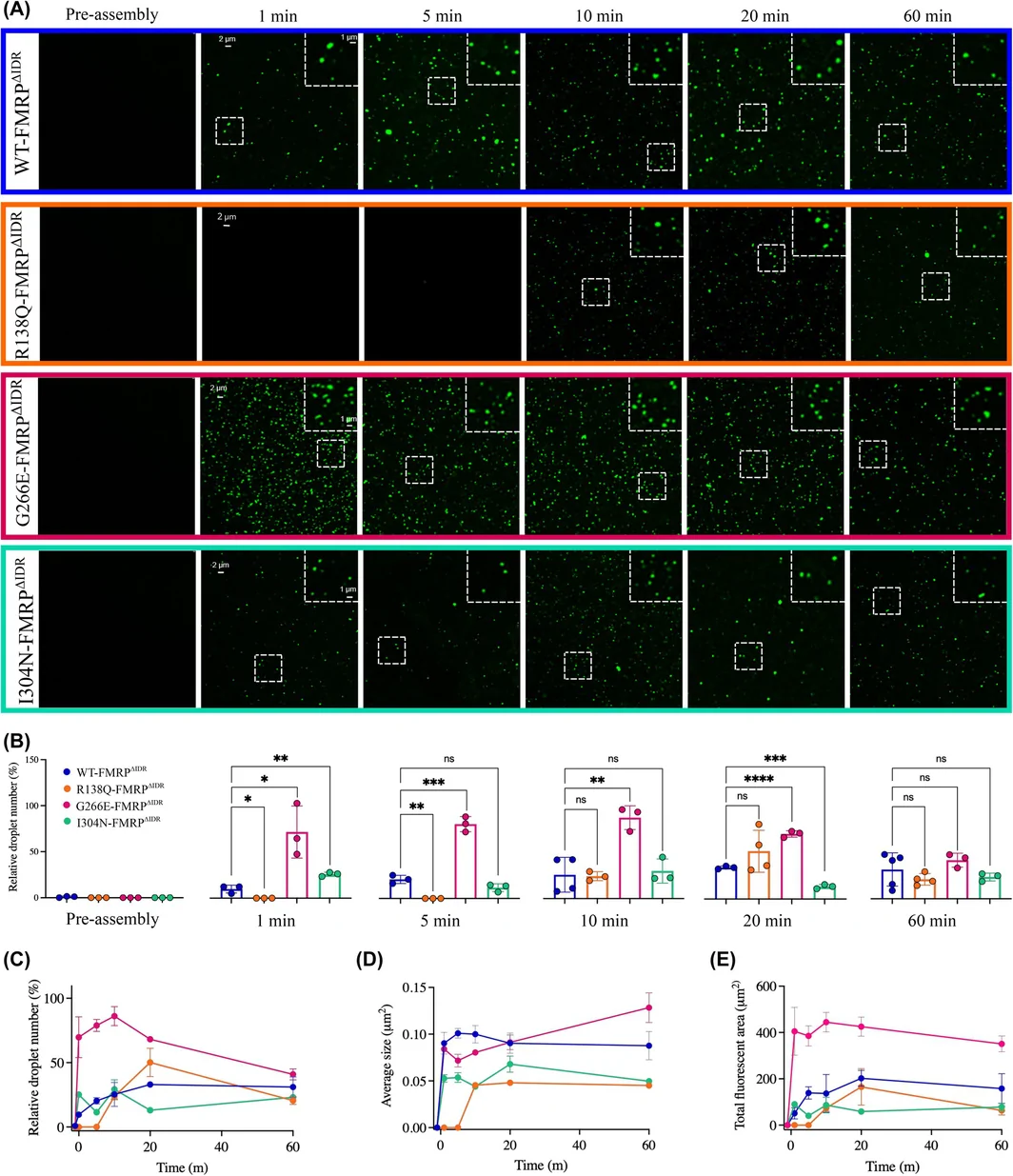
Time‐dependent droplet formation of FMRP^ΔIDR^ variants. (A) Representative confocal fluorescence microscopy images of WT‐FMRP^ΔIDR^ and its variants at 3 μm labeled with the Alexa Fluor 488 dye (1:10 dye:protein ratio) during self‐assembly over time at RT. Experiment was performed once (*n* = 1) due to limited sample availability and technical issues. Images were acquired at the indicated time points (±1 min). The top‐right square of each image indicates a zoomed area of the indicated box. (B, C) Quantification of droplet number over time for the FMRP^ΔIDR^ and its variants. Droplet counts were normalized to the maximum mean value across all conditions (set as 100%), with the minimum set to 0%. (D) Quantification of the average projected droplet area (μm^2^) over time for FMRP^ΔIDR^ and its variants. (E) Quantification of the total fluorescent area for FMRP^ΔIDR^ and its variants. All data represent mean ± SD of at least three confocal fields per condition. Statistical analysis was performed using unpaired two‐tailed *t*‐test; significance was defined as *P* < 0.05.

WT‐FMRP^ΔIDR^ rapidly formed round‐shaped droplets, consistent with previous observations (Fig. 2C). The number of droplets increased up to 20 min and then remained constant up to 60 min (Fig. 5B), with droplet size stabilizing shortly after formation; consequently, the total fluorescence area also increased with time following the increase of the first factor (Fig. 5C–E). A similar trend was observed for I304N‐FMRP^ΔIDR^, although the droplets formed were slightly smaller in size. In contrast, R138Q‐FMRP^ΔIDR^ droplets appeared only after approximately 10 min of incubation, indicating delayed condensation kinetics compared with the WT. The droplets were smaller and progressively decreased both in number and total area over time, suggesting a partial dissolution due to the dynamic exchange with the soluble protein pool (Fig. 5C–E). G266E‐FMRP^ΔIDR^ displayed the most distinctive behavior, characterized by the immediate formation of numerous droplets. Over time, their number decreased while their average size increased, with a nearly constant total droplet area, consistent with coalescence processes and/or solidification leading to the formation of larger condensates/aggregates without changes in total condensate mass (Fig. 5C–E).

Overall, these results demonstrate that each mutation differentially modulates the kinetics of FMRP^ΔIDR^ phase separation and the morphology of the resulting condensates, revealing mutation‐specific effects on condensate formation, stability, and maturation.

FRAP experiments performed at 5 μm concentration under the same phase separation conditions adopted for the WT protein highlighted further differences in the dynamic behavior of the droplets. Both R138Q‐FMRP^ΔIDR^ and G266E‐FMRP^ΔIDR^ formed round‐shaped droplets but also irregular‐shaped particles, likely resulting from incomplete coalescence events or dynamic exchange at the condensate interface between the condensed and soluble phases (Fig. 6A,B). Upon photobleaching, these assemblies exhibited only partial recovery of relative fluorescence intensity (~40% of the pre‐bleaching fluorescence), indicating a mixed liquid and gel‐like nature (Fig. 6D,E). In contrast, I304N‐FMRP^ΔIDR^ droplets showed high internal mobility (Fig. 6C), with rapid and complete fluorescence recovery, slightly higher than that of the WT‐FMRP^ΔIDR^ (Fig. 6D,E).

**Fig. 6:**
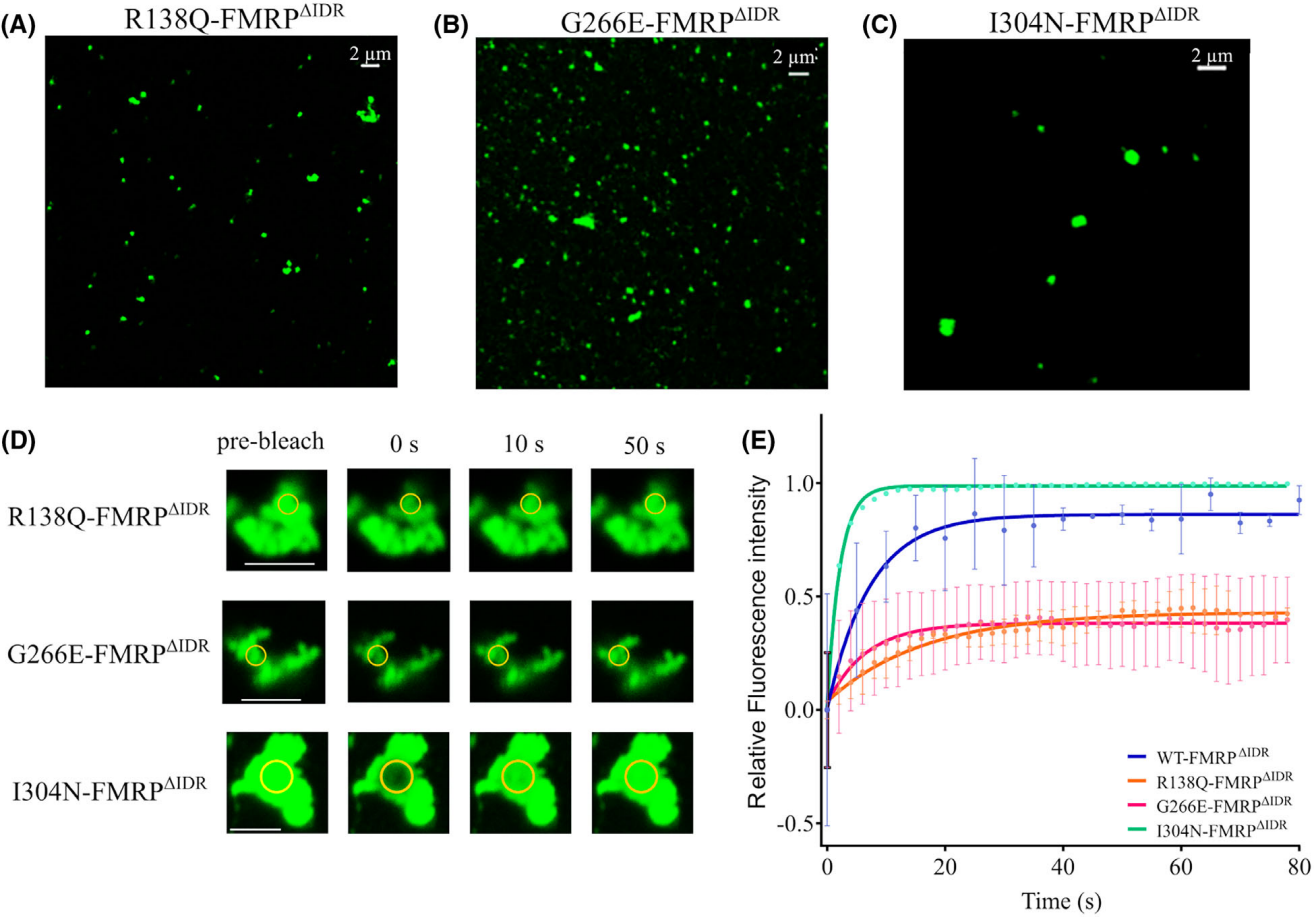
Dynamic behavior of droplets formed by FMRP^ΔIDR^ variants. (A–C) Representative (from *n* = 3 independent experiments) confocal fluorescence microscopy images of 5 μm Alexa Fluor 488‐labeled protein variants (1:10 dye:protein ratio) in self‐assembly buffer after mixing (D). Representative confocal microscopy images at different time points from the FRAP experiments of droplets formed by FMRP^ΔIDR^ variants. Scale bars: 2 μm. Yellow circles indicate the approximate bleached area; bleaching Regions of Interest (ROIs) were defined during acquisition and adapted to sample morphology. (E) FRAP recovery curves referred to droplets depicted in (D). Relative fluorescence intensity after photobleaching was plotted over time. Fluorescence values were normalized to pre‐bleach ones (taken as 1) and post‐bleach ones (taken as 0). Error bars: SD (*n* = 2 for R138Q‐FMRP^ΔIDR^, *n* = 3 for G266E‐FMRP^ΔIDR^, *n* = 1 for I304N‐FMRP^ΔIDR^). Relative fluorescence intensity after photobleaching versus time is also shown for comparison for the WT‐FMRP^ΔIDR^. It corresponds to the trace obtained at 5 μm and shown in Fig. 2.

In conclusion, FXS‐linked pathogenic mutations differentially affect droplet formation kinetics, number, size, and dynamics of FMRP^ΔIDR^, with the R138Q and G266E substitutions altering most parameters of condensate behavior, and the I304N mutation preserving the kinetics, number, and liquid‐like properties observed in the WT protein construct.

### FXS‐linked mutations alter the aggregation properties of FMRP^ΔIDR^

While examining the LLPS properties of FMRP^ΔIDR^ at a concentration of 10 μm in self‐assembly buffer, we observed the presence of insoluble aggregates (Fig. 2E). Previous studies have highlighted the intrinsic tendency of FMRP's N‐terminal region, up to the KH1 domain, to misfold and aggregate into β‐enriched structures [53, 54]. Here, we investigated whether the entire structured portion of FMRP (including KH2 and designated FMRP^ΔIDR^) can form amyloid fibrils under our conditions of analysis, which include the presence of macromolecular crowding.

Since the aggregates in Fig. 2E were observed using 10 μm FMRP^ΔIDR^ labeled with Alexa Fluor dye, we sought to determine whether this was also the case with unlabeled protein. Figure 7A shows a DIC image of unlabeled WT‐FMRP^ΔIDR^ at 10 μm and RT, revealing the presence of aggregates comparable to those observed with the labeled protein (Fig. 2E). To investigate their nature, we added to the pre‐formed aggregates an equimolar amount of Thioflavin‐T (ThT), a fluorescent dye commonly used for amyloid detection [63]. Under these conditions, we observed an immediate fluorescence signal (Fig. 7B), suggesting the fibrillar nature of these aggregates. To confirm their fibrillar morphology, we employed transmission electron microscopy (TEM) at a slightly higher protein concentration (20 μm) after 60 min of incubation at RT in the self‐assembly buffer, which revealed fibrillar structures with diameters of ~10 nm (Fig. 7C).

**Fig. 7:**
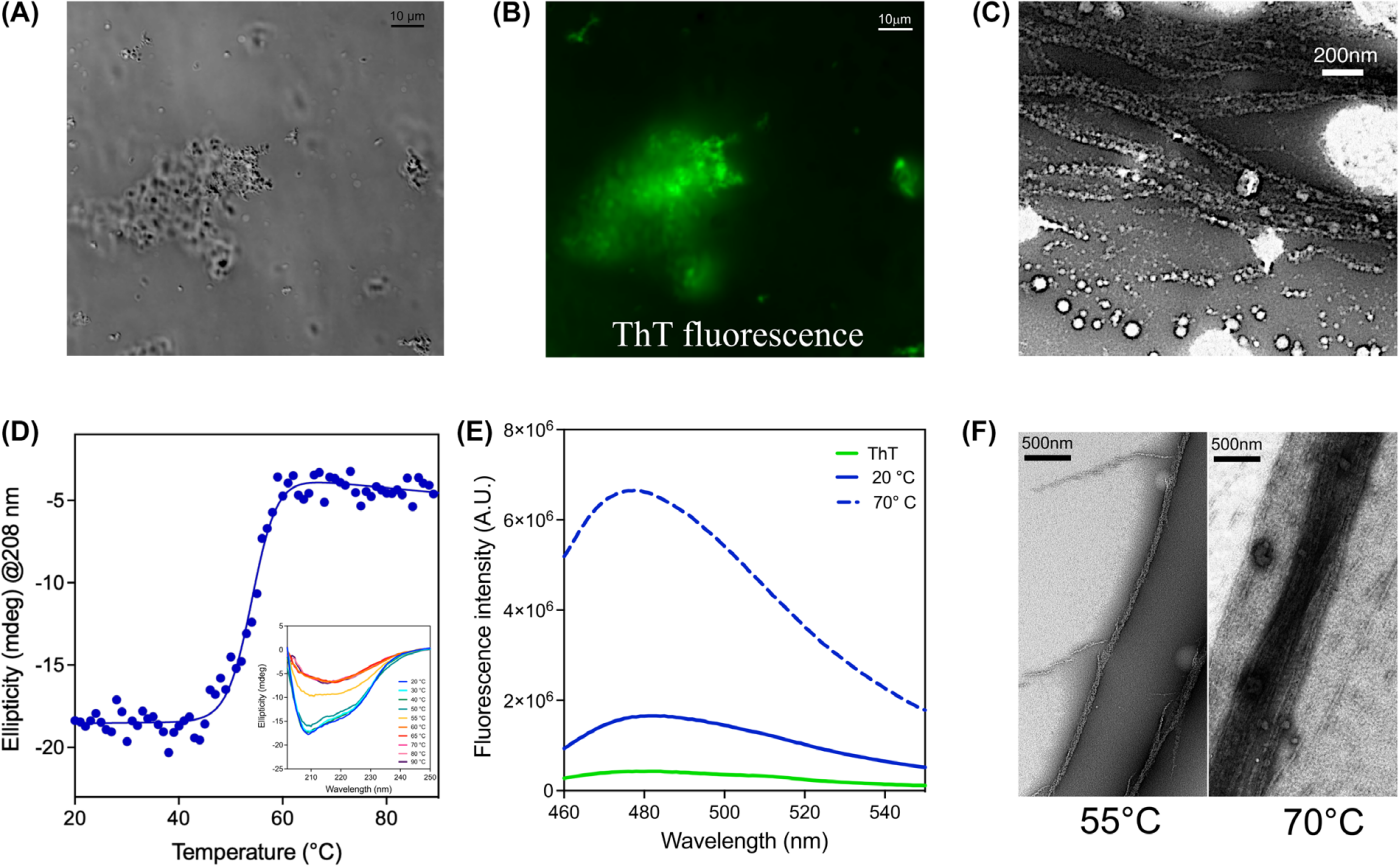
Fibrillogenic propensity of FMRP^ΔIDR^. (A) Representative (from *n* = 3 independent experiments) DIC image of 10 μm unlabeled WT‐FMRP^ΔIDR^ in self‐assembly buffer. (B) ThT fluorescence emission from the same aggregate shown in (A) recorded after addition of 10 μm ThT. (C) Representative (from *n* = 2 independent experiments) TEM analysis of 20 μm WT‐FMRP^ΔIDR^ after 60 min incubation in self‐assembly buffer at RT. (D) Thermally‐induced transition monitored by far‐UV CD at 208 nm for 8 μm WT‐FMRP^ΔIDR^ in 20 mm Tris/HCl (pH 7.4), 100 mm NaCl. Far‐UV CD spectra collected at different temperatures are shown in the inset. (E) ThT fluorescence emission spectra of 10 μm WT‐FMRP^ΔIDR^ in the same buffer as in panel D, collected after 10 min of incubation at either 20 °C (solid line) or 70 °C (dashed line) and mixed with an equimolar amount of ThT. The spectrum of ThT alone is shown in dark gray. (F) Representative (from *n* = 2 independent experiments) TEM analysis of fibrils formed by 10 μm WT‐FMRP^ΔIDR^ after 10 min of incubation at 55 °C and 70 °C in the same buffer as in panel D.

Furthermore, we examined whether high temperature could also promote FMRP^ΔIDR^ fibrillization. Far‐UV CD spectra were recorded at increasing temperatures in self‐assembly buffer lacking DTT and PEG 8 K to exclude LLPS induction at RT. At 20 °C the spectrum of FMRP^ΔIDR^ was consistent with a well folded α/β protein; however, as the temperature increased, an irreversible transition toward a β‐rich conformation was observed starting from ~50 °C (inset of Fig. 7D), with an estimated midpoint transition temperature of 54 ± 0.6 °C (Fig. 7D). This spectral change likely reflects a conformational transition from a α/β to an all β‐structure, rather than a simple thermal unfolding. Interestingly, ThT fluorescence assays performed on 10 μm FMRP^ΔIDR^ pre‐incubated under the same conditions at 70 °C for 10 min showed a marked increase in ThT fluorescence, indicating amyloid fibril formation (Fig. 7E); this result was further confirmed by TEM images at 55 °C and 70 °C under the same conditions, protein concentration and incubation time (Fig. 7F). It is worth noting that a significant, albeit lower, ThT fluorescence signal was detected in the self‐assembly buffer also in the absence of the crowding agent and DTT even at 20 °C (Fig. 7E, blue solid line), confirming the intrinsic tendency of the FMRP^ΔIDR^ to form fibrillar aggregates.

We next explored whether the concentration‐dependent transition from LLPS to amyloid is altered by the FXS‐linked mutants analyzed in this study. DIC analysis performed with unlabeled proteins showed that increasing the protein concentration to 10 μm in the self‐assembly buffer at RT led to the formation of large aggregates for the R138Q‐FMRP^ΔIDR^ variant (Fig. 8A, left panel). The binding of ThT to the pre‐formed aggregates of this variant supports their fibrillar nature (Fig. 8A, right panel), consistent with observations obtained with WT‐FMRP^ΔIDR^ (Fig. 7B). By contrast, no such aggregates were observed at this concentration for the G266E‐FMRP^ΔIDR^ and I304N‐FMRP^ΔIDR^ variants (Fig. 8B,C).

**Fig. 8:**
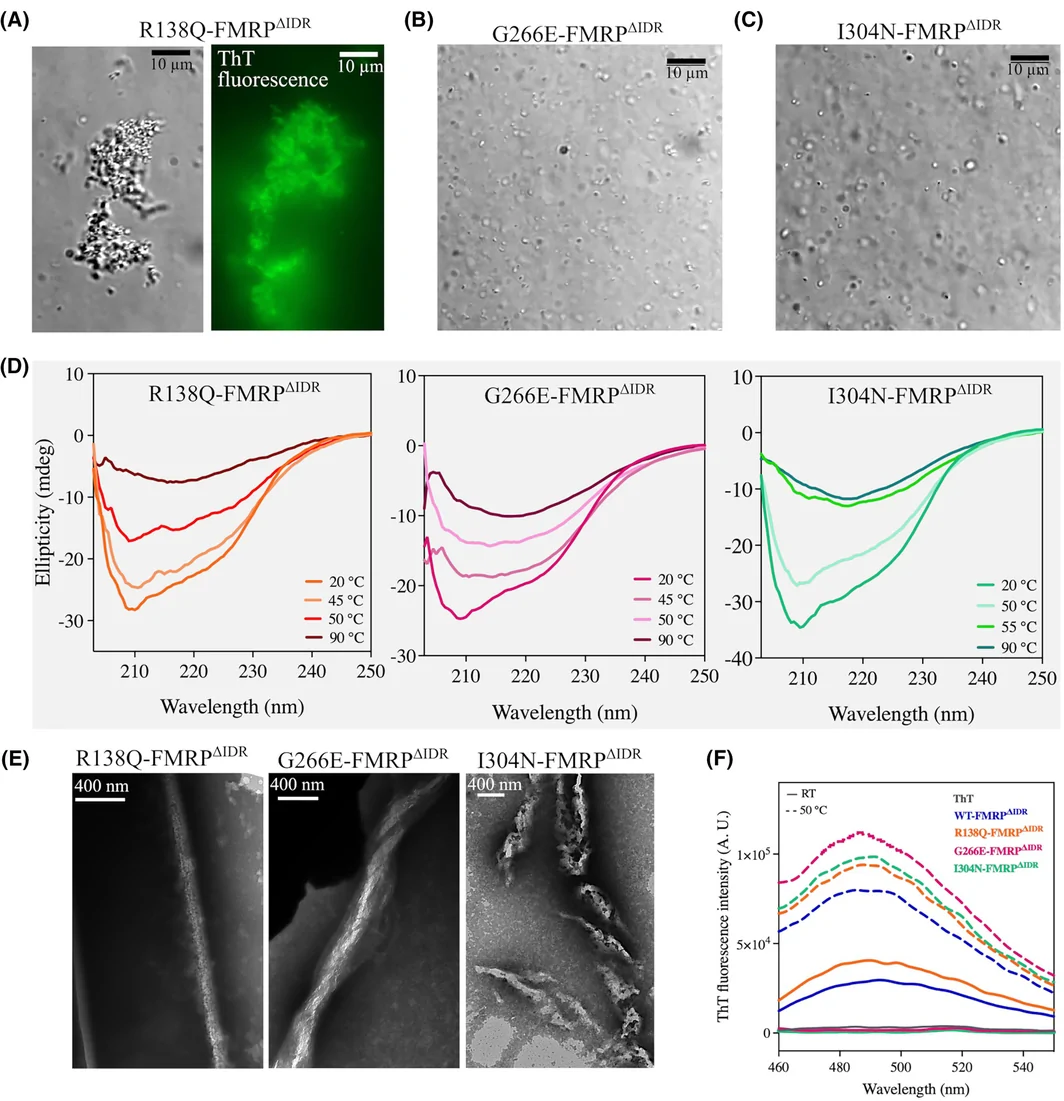
Amyloid formation propensity of FMRP^ΔIDR^ variants. (A–C) Representative (from *n* = 2 independent experiments) DIC images of 10 μm unlabeled protein mutants in self‐assembly buffer at RT. For the R138Q variant, the corresponding ThT fluorescence image of the same field, recorded after addition of 10 μm ThT, is also shown. (D) Far‐UV CD spectra of FMRP^ΔIDR^ variants recorded at increasing temperatures in 20 mm Tris/HCl (pH 7.4), 100 mm NaCl. (E) TEM images of FMRP variants (10 μm) after 10 min of incubation at 70 °C in the same buffer as in panel D. Experiment was performed once (*n* = 1) due to limited sample availability and technical issues. (F) ThT fluorescence emission spectra of 10 μm protein samples mixed with an equimolar amount of ThT in 20 mm Tris/HCl (pH 7.4), 100 mm NaCl, after 10 min incubation at RT (solid lines) or 50 min incubation at 50 °C (dotted lines).

Given that far‐UV CD spectroscopy revealed a temperature‐induced transition of WT‐FMRP^ΔIDR^ toward an amyloid‐like fibrillar structure under these conditions (Fig. 7D), we extended this analysis to the mutant proteins (10 μm protein, 20 mm Tris/HCl, pH 7.4, 100 mm NaCl). All three variants exhibited a similar α/β to β‐rich structural transition with increasing temperature, consistent with the formation of protein aggregates enriched with β‐sheet structure (Fig. 8D). TEM images on samples collected after the conformational transition induced by exposure of the proteins (10 μm) to 70 °C under the same conditions showed clear linear fibrils for R138Q‐FMRP^ΔIDR^, few and short fibrils for G266E‐FMRP^ΔIDR^, and no fibrillar morphology for I304N‐FMRP^ΔIDR^ (Fig. 8E). Notably, incubation of all variants at lower temperature (50 °C for 50 min) also resulted in a marked increase in ThT fluorescence (Fig. 8F, **dotted lines**); however, only the WT‐FMRP^ΔIDR^ and R138Q‐FMRP^ΔIDR^ variants showed a significant, albeit weak, increase in ThT fluorescence after 10 min at RT (Fig. 8D, **solid lines**). Taken together, these results demonstrate that WT‐FMRP^ΔIDR^ intrinsically tends to form amyloid‐like fibrils, a property that is enhanced by mimicking intracellular crowding conditions and thermal stress; such inherent tendency is partially retained by the disease‐associated variants with a ranking of WT ≈ R138Q >> G266E ≈ I304N at RT and WT ≈ R138Q > G266E > I304N at high temperature.

## Discussion

FMRP is a multidomain RNA‐binding protein highly expressed in neurons, containing RNA/protein interaction domains and a large C‐terminal intrinsically disordered region (IDR). These features confer high multivalency, predisposing FMRP to form highly ordered assemblies or biomolecular condensates when protein concentration exceeds a critical threshold [64, 65, 66, 67]. To date, *in vitro* studies have established the pivotal role played by the CTR of FMRP in phase separation, showing that this process can be modulated by RNA and/or target proteins and by post‐translational modifications, such as phosphorylation or methylation [26, 27, 28, 29, 30]. However, it has been shown that phase separation can also emerge from interactions between folded domains, via either homo‐ or heterotypic interactions [48, 66, 67]. These observations prompted us to explore whether the N‐terminal structured region (NTR) of FMRP devoid of the disordered region (FMRP^ΔIDR^, Fig. 1) retains condensation potential. This study was also prompted by observations that the NTR of FMRP has an intrinsic tendency to aggregate [53, 68] and that the KH1 and KH2 domains contribute to the formation and regulation of neuronal granule and their dynamics [34, 69].

Our *in vitro* data showed that FMRP^ΔIDR^ undergoes LLPS at low μm concentrations, at physiological pH and RT, under conditions mimicking cellular crowding, even in the absence of RNA or protein partners (Fig. 2). Upon increasing FMRP^ΔIDR^ concentration, the droplets lose sphericity and dynamicity (Fig. 2), evolving into large fibrillar aggregates as demonstrated by ThT fluorescence and TEM analysis (Fig. 7). Interestingly, a connection between these two aggregative‐related phenomena has also been observed in other RNA‐binding proteins, such as FUS and TDP‐43 [70, 71, 72, 73, 74]. A temperature–induced unfolding in the absence of crowding agents also promoted the transition of the WT‐FMRP^ΔIDR^ from the native state to the amyloid one (Fig. 7D). These results reinforce and complement those previously observed on a shorter construct of the structured region of FMRP (up to KH1), as well as FXR1P and FXR2P, which have an intrinsic tendency to form β‐rich structures [54]. Building on our recent finding that the isolated KH0 domain possesses an intrinsic fibrillogenic propensity [62], a key open question remains as to which structural element (or sequence) within FMRP drives fibrillogenesis. Further dissection is needed to determine whether the effect is confined to KH0 or involves additional domains (KH1, KH2, and Tudors).

A few missense mutations in the *fmr1* gene, leading to the expression of FMRP with altered function, have been correlated with FXS phenotypes [46]. Among these, the mutations R138Q, G266E, and I304N are localized in the three KH domains of FMRP, substantially impairing its function [23, 55, 75, 76, 77, 78]. A second aspect we examined in this study was whether these mutations could affect the *in vitro* condensation propensity of FMRP^ΔIDR^, as well as its fibrillar aggregation propensity. These mutations have been shown to have distinct effects on the folding, stability, and fibril‐forming propensity of individual KH domains [60, 61, 62], but their impact in the context of the full NTR had not been explored. Here, we found that their effects on the conformational stability of the entire FMRP^ΔIDR^ are less severe than those observed for the isolated KH domains. Specifically, R138Q‐FMRP^ΔIDR^ retains near‐native conformational stability (Fig. 3, Table 1), despite the modest destabilization observed in the isolated KH0 [62]. Moreover, neither G266E‐FMRP^ΔIDR^ nor I304N‐FMRP^ΔIDR^ fully unfolds the multidomain protein, although they both significantly decrease the stability of NTR (Fig. 3, Table 1). In particular, when studied in the context of the isolated domain, the G266E mutation causes complete unfolding of the KH1 [60]. A similar effect was observed when the I304N mutation was introduced at the equivalent topological position in the KH6 of Vigilin [61] or in the isolated KH2 domain (our unpublished data). These data indicate the presence of inter‐domain interactions that stabilize the overall NTD and buffer the deleterious effects of mutations observed in the isolated domains. Notably, it is known that the effect of the I304N mutation is also buffered when studied in the context of the KH1–KH2 tandem, where a destabilization is observed, yet the secondary structure is retained [55]. Although the full three‐dimensional structure of FMRP is not experimentally available, we can interpret our results based on the AlphaFold model, which predicts KH0 to be associated with the Tudor domains, while KH1, located centrally within the N‐terminal region (NTR), is connected to the Tudor‐KH0 cluster through a long α‐helix and arranged in tandem with KH2 (Fig. 1). According to this model, the three KH domains are tightly interconnected, suggesting that the effect of mutations in individual domains may be buffered in this structural context. In particular, KH1 may act as a structural hub essential for maintaining the multidomain interaction network, which could explain the more pronounced effect of the G266‐associated mutation compared with those in KH0 and KH2. However, the connecting α‐helix is predicted with low to medium confidence, leaving open the possibility that these regions may adopt multiple conformations.

What is the effect of these mutations on the propensity of FMRP^ΔIDR^ to undergo LLPS, and how do they influence the delicate balance between LLPS and aggregation? All FMRP^ΔIDR^ mutants underwent LLPS with similar apparent C*sat* values with respect to the WT (Fig. 4). Neither the WT protein nor the variants showed time‐dependent aggregation at 3 μm in the tested timeframe (Fig. 5A). However, differences emerged in droplet formation kinetics and dynamics: G266E‐FMRP^ΔIDR^ formed droplets faster and in greater number with a propensity to coalesce over time; R138Q‐FMRP^ΔIDR^ formed smaller and less stable droplets, with slower kinetics; I304N‐FMRP^ΔIDR^ mirrored WT behavior in terms of both rate of formation and number of droplets. To interpret these results, we have to take the net charges of the various FMRP domains into consideration. At the pH value of 7.4 at which our experiments have been carried out, the KH0 and all other four folded domains are positively and negatively charged, respectively, as the former (KH0, res: 125–200) has an isoionic point (pI) of 9.21 and all the others (TUD1, res: 4–50; TUD2, res: 63–115; KH1, res: 218–280; and KH2, res: 281–425, Δ331–396) have pIs below 5.80 including and excluding the linker regions pI value as calculated with the ProtParam application of Expasy® [79]. Electrostatic forces are a dominant factor in LLPS [80, 81, 82] and it is likely that the electrostatics of FMRP LLPS involve attractions between domains with opposite charges, as it was found for the TDP‐43 fragment containing the three folded domains [67]. The R138Q, G266E, and I304N mutations decrease the positive net charge of KH0 (pI from 9.21 to 8.82), cause a further increase of the negative net charge of the KH1 (pI from 5.12 to 4.93), and maintain an identical negative net charge of the KH2 (∆331–396, pI 5.15), respectively, providing an explanation for their different effects on LLPS kinetics and droplet density of the FMRP^ΔIDR^ construct studied here (decelerated, accelerated, and unvaried kinetics, respectively). Interestingly, in this context, FRAP experiments showed that only droplets from WT‐ and I304N‐FMRP^ΔIDR^ (at 5 μm) fully recovered the bleached fluorescence within 20 s (Figs 2 and 6E).

The role of the disordered 331–396 loop within the KH2 domain in this process remains to be clarified, as this loop is absent in our construct as it is in the FXR1 and FXR2 paralogs [4]. Given its intrinsically disordered nature, it could contribute to the protein condensation properties. Notably, the calculated pI of the KH2 domain including the 331–396 loop is 7.04, lower than that of the KH0 domain and consistent with previous considerations. Furthermore, it is worth noting that the KH2 loop and the C‐terminal disordered region are variable in the FMRP isoforms generated by alternative splicing [3], suggesting that their contribution to condensation may be isoform‐specific.

We next assessed whether these mutations alter the intrinsic propensity of the protein to undergo fibrillar aggregation and how they influence the liquid‐to‐amyloid transition. All FMRP^ΔIDR^ mutants, similarly to the WT protein, formed β‐sheet containing aggregates upon a temperature‐induced conformational transition (Fig. 8D,E). The fibrillar texture is evident for the WT and R138Q variants, less for the G266E mutant and very weak for the I304N mutant (Fig. 8E). At RT, an increase in ThT fluorescence was detected only for the WT and R138Q variants (Fig. 8F), suggesting that the G266E and I304N mutations reduce fibrillization propensity under milder conditions. This observation is supported by DIC analysis, showing that at the highest concentration tested (10 μm), only the WT and R138Q variants formed large ThT‐positive aggregates (Figs 7A–C and 8A–C), indicating a significantly higher propensity for fibrillar assembly under crowding‐like conditions. This behavior is consistent with previous observations for the isolated KH0 domain [62]. Altogether, these data indicate that only the R138Q variant maintains a fibrillar aggregation profile similar to that of the WT protein, whereas the other two mutations largely attenuate amyloid aggregation in the tested conditions at RT, with or without crowding agents. Since the fundamentals of amyloid fibril formation and associated mutational effects are known in more detail [83, 84, 85, 86, 87, 88], we can rationalize these results considering the physicochemical changes caused by the three mutations at their corresponding sites at the molecular level. The G266E and I304N mutations both involve a large decrease in hydrophobicity, a large decrease of β‐sheet propensity (the latter mutation), and an exacerbation of the negative net charge (the former mutation). All these changes are expected to largely decrease the aggregation propensity [83, 84, 85, 86, 87, 88], as observed experimentally. By contrast, the R138Q mutation causes a decrease in hydrophilicity and an increase of the negative net charge of the protein, two opposite effects that cancel each other, again as observed experimentally.

In conclusion, we demonstrate that under *in vitro* conditions mimicking the crowded cellular environment, the N‐terminal structured region of FMRP containing the five structured domains can undergo LLPS and aggregation. These findings underscore the synergistic roles of both structured and unstructured regions in FMRP and call for further investigation. In neurons, FMRP normally cycles between a translation‐competent state and a condensate‐associated repressed state, and regulation of condensate dynamics by the C‐terminal IDR has been shown to tune activity‐dependent translational control [28]. KH domains further modulate this process by increasing RNA‐dependent valence and stabilizing granule assembly/transport, and the FXS‐linked KH mutations alter this balance [34]. We provide here evidence that the R138Q, G266E, and I304N FXS‐linked mutations have different effects on protein conformational stability, LLPS and fibrillogenic propensity, highlighting the crucial role of the KH domains in these processes (Fig. 9). In summary, the mutations either: (i) destabilize the NTR (G266E/I304N), (ii) change remarkably the kinetics of droplet formation through LLPS and suppress their liquid nature (R138Q/G266E), or (iii) promote solid or gel‐like aggregation in a form distinct from amyloid that may be more deleterious (G266E, I304N) (Fig. 9). Although these effects alone cannot explain the complex pathogenesis of FXS, they provide mechanistic explanations into how KH‐domain mutations exert their pathogenic mechanism of action. Indeed, it is well known that (i) destabilization of folded domains predisposes them to misfolding and aggregation, (ii) that low FRAP recovery within droplets indicates a rapid liquid‐to‐solid transition, and (iii) protein aggregates distinct from amyloid can be deleterious and cause neuronal dysfunction. All three mutations alter at least one or more factors in the pathogenic direction and none of them rather point to the beneficial direction, explaining why they can be pathogenic. Moreover, our observations suggest that the complex equilibrium between protein domain stability, LLPS, and fibrillar aggregation should be increasingly considered in future physio‐pathological studies on FMRP.

**Fig. 9:**
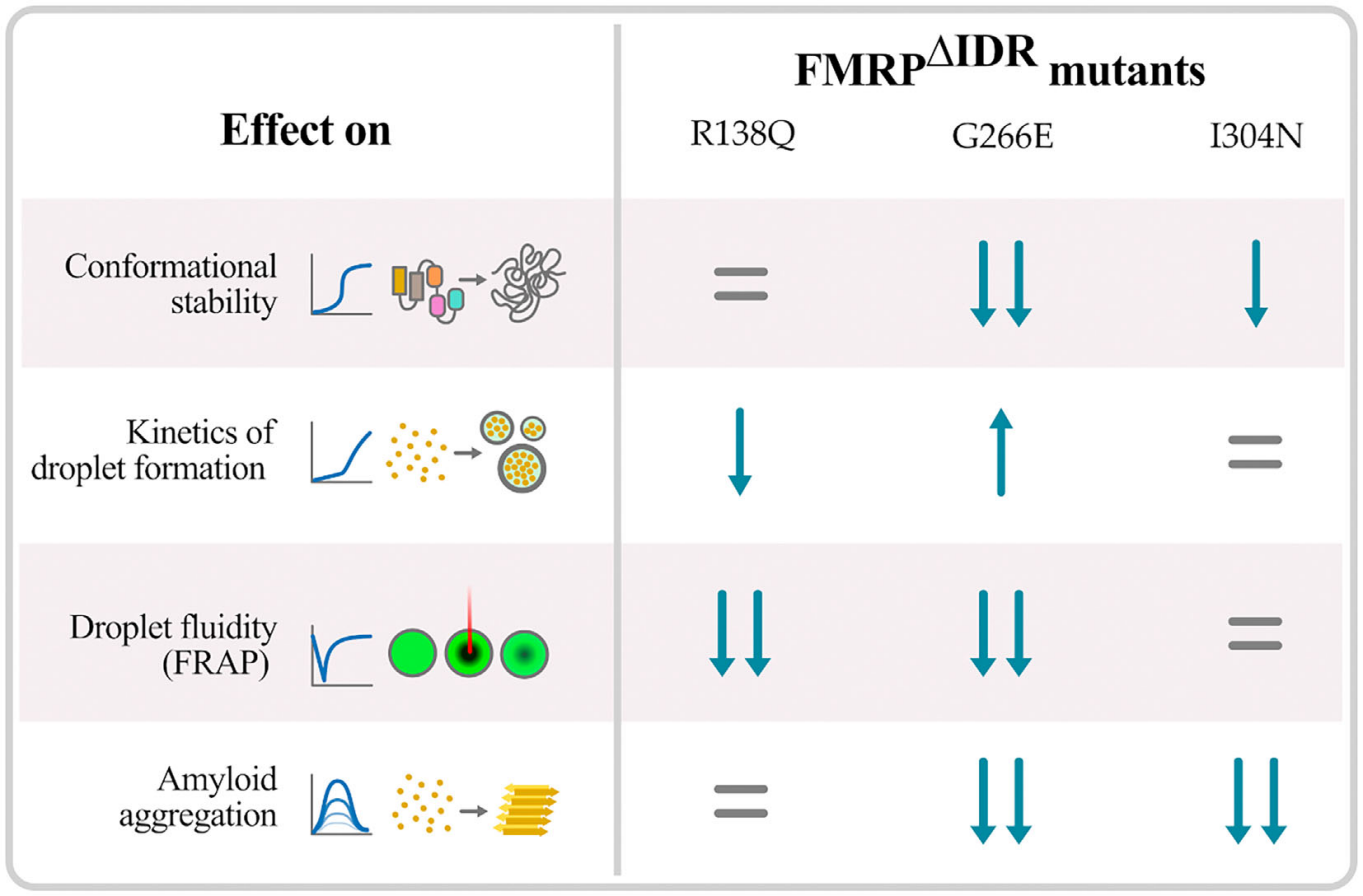
General summary of the effect of different mutations of FMRP^ΔIDR^ with respect to the WT‐ FMRP^ΔIDR^ on conformational stability, kinetics of droplet formation, droplet fluidity, and amyloid aggregation propensity. Single arrows indicate a less pronounced effect, whereas double arrows indicate a more pronounced effect.

## Materials and methods

### Protein expression and purification

A truncated construct of the canonical isoform of human FMRP (UniProt: Q06787‐1), named FMRP^ΔIDR^ (residues 1–425 with a deletion encompassing the variable loop in KH2, res: 331–396), and three FXS‐linked variants (R138Q‐FMRP^ΔIDR^, G266E‐FMRP^ΔIDR^, I304N‐FMRP^ΔIDR^) were cloned into a pET28a+ vector with a His‐tag fused to the N‐terminus (sequence MGSSHHHHHHSSGLVPRGSH preceding the first residue Met1).

All mutants were generated by site‐directed mutagenesis using the QuikChange Lightning Site‐Directed Mutagenesis kit (Agilent Technologies, Santa Clara, CA, USA). Primers were purchased from Eurofins Genomics, and all sequences were verified by DNA sequencing.

Proteins were expressed in *Escherichia coli* BL‐21 (DE3) cells grown in LB broth supplemented with 34 μg·mL^−1^ kanamycin at 37 °C. Protein expression was induced at an OD600 of 0.6–0.7 with 0.3 mm Isopropyl‐β‐D‐1‐thiogalactopyranoside (IPTG), followed by incubation at 22 °C for 4 h. Cells were harvested by centrifugation. Pellets were resuspended in lysis buffer containing 20 mm Tris/HCl (pH 7.4), 300 mm NaCl, 5% glycerol, and a protease inhibitor (Complete EDTA‐free, Roche, Basel, CH). Cells were lysed by sonication, and the lysates were cleared by centrifugation.

The supernatant was supplemented with 25 mm imidazole and loaded onto a HisTrap FF column (GE Healthcare, Chicago, IL, USA) equilibrated with the same buffer. Proteins were eluted with a linear imidazole gradient (20 mm to 1 mM). Eluted fractions were diluted in 20 mm Tris–HCl (pH 8.0). The solution was applied to a HiTrap Q FF column (GE Healthcare) and eluted with a NaCl gradient (20 mm to 1 m NaCl). Collected fractions were buffer‐exchanged into 20 mm Tris–HCl (pH 7.4), 100 m NaCl using a HiTrap Desalting column (GE Healthcare). Final yields were approximately 5 mg·L^−1^ for FMRP^ΔIDR^, ~2.5 mg·L^−1^ for R138Q‐FMRP^ΔIDR^ and I304N‐FMRP^ΔIDR^, and ~1.5 mg·L^−1^ for G266E‐FMRP^ΔIDR^. Protein purity was confirmed by SDS/PAGE. Purified proteins were flash‐frozen in liquid nitrogen and stored at −80 °C. Before use, aliquots were thawed on ice and centrifuged at 16 200 ***g*** for 10 min at 4 °C to remove precipitate. Protein concentration was accurately determined and re‐verified prior to each spectroscopic measurement using the same theoretical extinction coefficient for all proteins calculated with ProtParam of Expasy® [79] (33 140 m ^−1^ cm^−1^) and confirmed by a Bicinchoninic Acid Assay (BCA) (ThermoFisher Scientific).

### Circular dichroism experiments

Far‐UV CD experiments were carried out using a Jasco J‐710 instrument (Jasco Inc.) equipped with a Peltier temperature control system. Far‐UV CD spectra (200–250 nm) were collected using protein concentrations of 12 μm for FMRP^ΔIDR^, R138Q‐FMRP^ΔIDR^, I304N‐FMRP^ΔIDR^, and 10 μm for G266E‐FMRP^ΔIDR^, in a buffer containing 20 mm Tris/HCl (pH 7.4) and 100 mm NaCl, at 20 °C, using a 1 mm quartz cuvette. Measurements were acquired at a scanning speed of 100 nm/min, and spectra represent the average of three acquisitions. Thermal denaturation profiles were monitored at 208 nm under the same conditions over a temperature range from 20 °C to 90 °C, with a temperature increment of 1 °C/min.

Equilibrium chemical denaturation experiments were performed using urea as a denaturating agent. Protein concentrations were 10 μm FMRP^ΔIDR^, G266E‐FMRP^ΔIDR^, and I304N‐FMRP^ΔIDR^, and 6 μm for R138Q‐FMRP^ΔIDR^. CD signals at 222 nm were plotted as a function of urea concentration, and the data were fitted using a two‐state equation:

$$\;\text{Yobs}=\frac{\left(Y_{N}+\left(m_{N}*D\right)\right)+\left(\left(Y_{D}+\left(m_{D}*D\right)\right)*\mathrm{ex}\left(\frac{m_{\mathrm{DN}}*\left(D-D_{\frac{1}{2}}\right)}{\mathrm{RT}}\right)\right)}{1+\exp\left(\frac{m_{\mathrm{DN}}*\left(D-D_{\frac{1}{2}}\right)}{\mathrm{RT}}\right)}$$

where *Y*_*N*_ and *Y*_*D*_ refer to the y‐intercepts and *m*_*N*_ and *m*_*D*_ represent the slopes of the native and denatured baselines, respectively; *D* is the denaturant concentration. *R* is the gas constant and *T* the experimental temperature (K). Δ*G*_*DN*_ was calculated using the equation: Δ*G* ^0^_*DN*_ = *m*_*DN*_ * *D*_1/2_ where *m*_*DN*_ is the slope of the denaturation curve in the transition region and *D*_1/2_ is the denaturant concentration at the midpoint of the denaturation.

### Turbidimetric assay

Turbidimetry measurements were performed using a SpectraMax® Mini multi‐mode microplate reader in clear bottom 96‐well plates (Nunc™ Microwell™). Self‐assembly of FMRP^ΔIDR^ and its mutants was initiated by diluting protein samples to various concentrations in a self‐assembly buffer containing 20 mm Tris/HCl (pH 7.4), 100 mm NaCl, 2 mm DTT, 10% (w/v) PEG 8 K at room temperature (RT). Optical density at 400 nm (OD_400_) was measured to monitor turbidity. All measurements were performed in triplicate and averaged. The apparent saturation concentration (C*sat*) was determined from the intersection point of two straight lines used to fit the experimental data. Measurements were performed up to 7.5 μm, which was sufficient to define the apparent Csat while minimizing protein consumption and ensuring that the analysis was carried out within the linear range of the assay.

### DIC and ThT fluorescence microscopy

Unlabeled protein samples (2.5 or 10 μm FMRP^ΔIDR^, 10 μm FMRP^ΔIDR^ mutants) were mixed with the self‐assembly buffer (20 mm Tris/HCl (pH 7.4), 100 mm NaCl, 2 mm DTT, 10% (w/v) PEG 8 K) at RT and spotted onto a μ‐Slide 18‐well glass bottom plate (Ibidi GmbH). DIC images were acquired using a Crest X‐Light V3 confocal spinning disk microscope (Nikon, Europe B.V., secondary office, Campi Bisenzio, Florence, Italy) equipped with a Kinetix sCMOS camera (Teledyne Photometrics, Waterloo, Ontario, Canada), using a 60x (oil immersion, N.A. 1.4) objective. When present, aggregates were stained with 10 μm Thioflavin T (ThT; Merck, kGaA, Darmstad, Germany). DIC and ThT fluorescence images (FITC channel) were acquired using a multi‐LED light source (D‐LEDI, Nikon). All images were acquired using NIS‐Elements AR software (version 5.42.05, Nikon).

### Confocal fluorescence microscopy and FRAP measurements

FMRP^ΔIDR^ and its mutants were labeled with Alexa Fluor 488 (AAT Bioquest) by incubating proteins (10 or 20 μm) overnight at 4 °C under agitation with a 1:10 dye:protein molar ratio, in 20 mm Tris/HCl (pH 7.4), 100 mm NaCl, and 2 mm DTT. Phase separation was induced by diluting labeled proteins to final concentrations of 3, 5, and 10 μm in self‐assembly buffer at RT. Immediately after dilution, 40 μL of the samples were spotted into a well of a μ‐Slide 18‐well glass bottom plate (Ibidi GmbH). FRAP experiments were performed as previously described [67] with minor modifications. Measurements were conducted within the first 15 min from dilution in self‐assembly buffer using the LAS AF FRAP Application Wizard on a Leica TCS SP8 × 3 microscope equipped with a Leica HC PL APO CS2 × 100/1.40 oil White objective and a 488‐nm laser at a scan speed of 700 Hz. Two pre‐bleaching images were acquired before photobleaching, which was carried out on selected regions of interest (ROIs) using 100% laser intensity for 20 s. Fluorescence recovery was recorded every 5 or 2 s for up to 80 s. Fluorescence recovery values were normalized to those of pre‐bleaching (set as 1) and post‐bleaching (set as 0).

For the time‐dependent droplet formation analysis, self‐assembly was induced by diluting Alexa Fluor 488‐labeled proteins to 3 μm into self‐assembly buffer. The samples were incubated in microcentrifuge tubes and, at defined time points (1, 5, 10, 20, 60 min), gently resuspended; then, 5 μL of each sample were spotted on a clean microscope slide, covered with a 18 mm glass coverslip, and visualized using a TCS SP8 scanning confocal microscopy system (Leica Microsystems) equipped with an argon laser source, under fluorescence mode as reported previously [59] with minor modifications. Alexa Fluor was excited at 488 nm and fluorescence emission was collected between 493 and 516 nm. Sample preparation and imaging required about 1 min, a standardized and controlled timeframe that allowed reproducibility and accurate analysis of the assemblies before any detectable evolution could occur. The average projected area and the number of the green fluorescent droplets were quantified using imagej [89] and the ‘Analyze Particles’ function, following background subtraction and the application of a constant threshold across all analyzed images. Data were expressed as means ± SD. Comparisons between the different groups were assessed using an unpaired two‐tailed t‐test. Statistical significance was determined at *P* < 0.05.

### ThT fluorescence assay

ThT was added to proteins pre‐incubated for 10 min at 20 °C and 70 °C (WT‐FMRP^ΔIDR^) and 10 min at RT or 50 min at 50 °C (WT‐FMRP^ΔIDR^ and its variants). Final ThT and protein concentrations were 10 μm. Experiments were carried out in 20 mm Tris/HCl pH 7.4, 100 mm NaCl. ThT fluorescence emission spectra were recorded between 460 and 550 nm (*λ*_*ex*_ = 440 nm) using an FL 6500 Fluorescence Spectrometer (PerkinElmer, Inc., Shelton, Connecticut, USA) or a FluorMax‐4‐spectrofluorometer (Jobin Yvon, Edison) using a 1 cm path‐length quartz cuvette at 20 °C.

### Transmission electron microscopy (TEM)

TEM analyses were performed using a CM 100 transmission electron microscope (Philips) equipped with a tungsten filament and operating at 100 kV. Specimens were prepared by negative staining as previously described [62]. Images were acquired at different magnifications using a CMOS PHURONA camera (Emsis) with a resolution of 3008 × 4112 pixels. For PEG‐induced aggregation, WT‐FMRP^ΔIDR^ was diluted to 20 μm in 20 mM Tris/HCl (pH 7.4), 100 mm NaCl, 2 mm DTT and 10% PEG 8 K. After 1 h of incubation at RT, the sample was centrifuged for 15 min at 11 400 ***g*** and 5 μL of the supernatant was used to resuspend the pellet. Then, 5 μL of the resuspended sample was applied onto a 200‐mesh carbon‐coated copper grid and allowed to absorb for 30 s. Excess liquid was removed by touching the edge of the grid with Whatman filter paper. The grid was briefly (1–2 s) washed with buffer lacking PEG 8 K, then dropped downwards onto 50 μL of commercial Nano‐W stain for 30 s. Excess stain was removed, and the grid was dried under vacuum before being imaged using TEM.

For temperature‐induced aggregation, 10 μm of FMRP^ΔIDR^ was incubated in a waterbath for 10 min at both 55 °C or 70 °C in 20 mm Tris/HCl, pH 7.4, 100 mm NaCl. FMRP^ΔIDR^ variants (10 μm) were incubated in a water bath for 10 min at 70 °C in the same buffer. Samples were centrifuged at 6800 ***g*** for 5 min, and 5 μL of each supernatant was applied onto a 200‐mesh carbon‐coated copper grid, allowed to absorb for 30 s or 1 min, the excess of sample was removed by touching the grid side with Whatman filter paper, and the grid was washed for 1–2 s with buffer before dropping it downwards on 50 μL of commercial Nano‐W stain for 30 s. Then, the excess of stain was removed, and the grid was dried under vacuum before TEM imaging.

## Conflict of interest

The authors declare no conflict of interest.

## Author contributions

AB: formal analysis, investigation, visualization. FA: supervision, validation, writing – review & editing. FlC: conceptualization, formal analysis, investigation, visualization, writing – original draft. ADM: conceptualization, funding acquisition, project administration, supervision, validation, writing—original draft. FG: investigation, visualization. GG: supervision, validation, visualization, writing—review & editing. IAA: formal analysis, investigation. GP: investigation. FT: formal analysis, investigation, visualization. CTA: conceptualization, funding acquisition, project administration, supervision, validation, writing—original draft. FaC: conceptualization, project administration, supervision, validation, writing—original draft.

## Data Availability

Data available on request from the authors.
